# From Adjunct to Essential: Integrating Supportive Care into Oncology

**DOI:** 10.3390/curroncol33060373

**Published:** 2026-06-22

**Authors:** Gilla K. Shapiro, Fredrick D. Ashbury, Jonathan Avery, Jacqueline L. Bender, Sylvie Lambert, Madeline Li

**Affiliations:** 1 Department of Supportive Care, Princess Margaret Cancer Centre, University Health Network, Toronto, ON M5G 2M9, Canada; jackie.bender@uhn.ca (J.L.B.); madeline.li@uhn.ca (M.L.); 2 Department of Psychiatry, Temerty Faculty of Medicine, University of Toronto, Toronto, ON M5T 1R8, Canada; 3 Dalla Lana School of Public Health, University of Toronto, Toronto, ON M5T 3M7, Canada; 4 Institute of Medical Science, University of Toronto, Toronto, ON M5S 3H2, Canada; 5 Clinical & Scientific Division, VieCure, Denver, CO 80111, USA; 6 Department of Internal Medicine—Medical Oncology, The Ohio State University, Columbus, OH 43210, USA; 7 Department of Oncology, University of Calgary, Calgary, AB 72N 4N1, Canada; 8 Department of Cancer Control Research, BC Cancer, Vancouver, BC V5Z 1L3, Canada; jonathan.avery@bccancer.bc.ca; 9 Ingram School of Nursing, McGill University, Montreal, QC H3A 2M7, Canada; sylvie.lambert@mcgill.ca; 10 St. Mary’s Research Centre, Montreal, QC H3T 0A2, Canada

## 1. From Psychosocial Oncology Evidence to Implementation

Psychosocial oncology is a discipline concerned with the social, psychological, emotional, spiritual, quality-of-life, and practical aspects of cancer for patients and families, with the aim of supporting whole-person care [1]. Importantly, this scope extends beyond patients alone, also encompassing caregivers, communities, providers, services, and system-level factors. Over the past several decades, the field has moved from advocacy for recognition toward consolidation of a robust empirical base demonstrating that psychosocial and supportive care interventions across the cancer continuum improve quality of life, symptom control, and the patient experience; mitigate financial toxicity; and improve health system outcomes [2]. In some contexts, they have also been associated with cancer survival [3]. Yet despite guidelines recommending routine psychosocial assessment and intervention [4,5,6,7,8,9], implementation remains uneven, fragmented, and vulnerable to structural constraints [10]. As we look ahead, the next decade of psychosocial oncology will likely be defined as much by implementation as by innovation, not only by the generation of new evidence but also by how effectively that evidence is implemented within routine cancer care systems, including a redefinition of human resources and technology solutions.

The Canadian Association of Psychosocial Oncology (CAPO) was established in 1987, and as the fourth decade of advancing psychosocial oncology in Canada approaches, this *Current Oncology* Special Issue, “Building Hope for the Next Decade of Psychosocial Oncology: Optimizing Integration of Supportive Care into Oncology Care”, was conceived to reflect this moment of transition. The Special Issue welcomed contributions spanning clinical practice, education, health systems, and policy research, including diverse perspectives related to inclusion, diversity, equity, and access. Collectively, the papers suggested that psychosocial oncology is poised to move beyond current practices and models of care, pointing instead toward a future characterized by greater integration within oncology practice.

Across the papers, contributors consider how supportive care can be better integrated into standard oncology practice in ways that are sustainable, equitable, and responsive to diverse patient, family, and caregiver needs. In cancer care, hope can be cultivated through systems that ensure timely access to appropriate, person-centered care that supports individuals in buffering stress, overcoming adversity and burnout, coping with unique challenges, and optimizing health, healing, and growth. The six papers included in this Special Issue collectively demonstrate multiple complementary pathways through which psychosocial oncology can move toward more integrated, responsive, and data-informed practices.

## 2. Precision and Pragmatism in Distress Screening

Routine screening for distress has been widely endorsed as a standard of oncology care [11]. In practice, however, implementation remains inconsistent, often due to concerns about the workflow burden and downstream capacity. Gascon et al. conducted a retrospective study of 172 patients with cancer in Toronto, Canada, examining a two-step sequential screening algorithm. For depression, an Edmonton Symptom Assessment System (ESAS) depression item score greater than 2 was followed by administration of the Patient Health Questionnaire-9 (PHQ-9) using a cutoff score of 15 or higher. For anxiety, an ESAS anxiety item score greater than 2 was followed by the administration of the Generalized Anxiety Disorder-7 (GAD-7) using a cutoff score of 15 or higher. The authors found that sequential screening significantly improved diagnostic accuracy for depression but not anxiety. These findings contribute practical evidence that balances diagnostic precision and clinical feasibility, prioritizing accurate case identification while minimizing unnecessary clinical burdens on patients and clinicians.

The results also suggest that screening for depression and anxiety may require distinct strategies rather than one-size-fits-all protocols. The absence of improved detection for anxiety may reflect a relatively low prevalence of generalized anxiety disorder in the sample, limited statistical power, or the heterogenous presentation of anxiety among patients with cancer. Nevertheless, this work has important implications for program design and reinforces the need for ongoing evaluation of screening practices using real-world clinical data. As cancer centers continue to face increasing patient volumes alongside constrained psychosocial resources and complex technology infrastructure, studies such as this one provide actionable guidance on how screening tools can be used more strategically. The authors also point toward the potential future role of artificial intelligence-augmented approaches to screening, which may offer new opportunities for personalizing psychosocial assessment and informing accurate diagnosis and deployment of stepped-care pathways.

## 3. Improving Services Through System Design

Two studies in this Special Issue highlight the need for intentional alignment across clinical workflows, professional roles, and organizational priorities. The Alberta Cancer Exercise (ACE) study by Shallwani et al. offers a compelling example. Among 306 participants with advanced cancer, participation in a 12-week community-based exercise program in Alberta, Canada, was associated with significant improvements in physical activity, physical fitness, and quality of life, with a very low rate of adverse events. The overall attendance rate in the program was 73.6%, with few participants withdrawing prior to program completion (*n* = 32). The ACE program involved full-body, group-based circuit training or personal training, with a combination of aerobic, resistance, balance, and flexibility exercises, delivered as 60 min or longer sessions of mild- to moderate-intensity exercise twice weekly for twelve weeks. These findings demonstrate that patients diagnosed with advanced cancer are willing and able to access, participate in, and benefit from exercise programs. Moreover, subgroup analyses highlighted differential effects, which reinforces the importance of tailoring supportive care interventions to the clinical context and patient need.

Complementing these patient-level findings, a qualitative study by Finless et al. examined healthcare provider experiences with an exercise referral pathway. Exercise for Cancer to Enhance Living Well (EXCEL) is an exercise intervention combining aerobic and resistance training. In qualitative interviews with 13 healthcare providers from Nova Scotia and Alberta, participants described greater confidence and willingness to refer patients to exercise oncology programs. They also identified important implementation barriers and considerations, including limited understanding of the roles of exercise professionals by healthcare providers and other oncology certified professionals within cancer care, the need for more streamlined and time-efficient referral processes, and the importance of receiving feedback about patients’ experiences and outcomes following referral.

These two exercise oncology papers illustrate how supportive care interventions can be operationalized within real-world cancer systems, ranging from coordinated (but parallel) community programs to more intentionally integrated referral pathways. While the effectiveness and safety of exercise interventions are established, these studies highlight that their sustainability depends on system-level factors such as interprofessional collaboration, clear role delineation, and reducing administrative burdens.

## 4. Educating the Public About Supportive Care

Public misperceptions about supportive care services, particularly palliative care, continue to contribute to stigma and delayed access. Biondo et al.’s paper on the development of the Understanding Palliative Care module in Calgary, Alberta, responds directly to this challenge. This publicly accessible, online educational module was informed by lived experience and integrates patient and caregiver narratives alongside evidence-based content. In doing so, the module reframes palliative care as comprehensive support delivered across the cancer trajectory rather than as care reserved for the end of life.

Feedback on the module emphasized the importance of using plain language, focusing on positive and clarifying messages rather than a myth-busting approach, featuring Albertans with lived experience, and the use of engaging graphic design. At the time of publication, the authors reported that web analytics indicated more than 3000 page visits, and early survey data suggested high user satisfaction. The collaborative development approach reinforces the ethical and practical importance of engaging patients and caregivers as partners in shaping how psychosocial and palliative care services are understood. This work also reflects a growing recognition that public-facing education is a critical strategy for normalizing and integrating supportive care into oncology care.

## 5. Addressing Fragmentation in Transition to Cancer Survivorship

As cancer treatments continue to improve with more novel therapies (e.g., through radioligand therapy), psychosocial oncology must increasingly attend to survivorship across the lifespan, including critical transitions that are often under-resourced and poorly coordinated. Carrier et al.’s report on a pilot study of transition readiness workshops for survivors of pediatric brain tumor (age 14 or older) and their parents in Montréal, Québec, addresses a population for whom long-term effects can include neurocognitive challenges, physical sequelae, dependency, and difficulties navigating adult-oriented healthcare systems. The intervention comprised three workshops, delivered by a multidisciplinary team, and focused on self-management of the disease, social skills and peer relationships, as well as cognitive challenges and return to daily activities.

However, only 12 survivor–parent dyads participated, representing a 38% recruitment rate, which reflects challenges frequently reported in pediatric survivorship research. There was high initial participation (83%) and high satisfaction (73%), but retention declined to 50% by the third workshop. Despite its modest scale, this study offers valuable insights into both the feasibility and constraints of conducting family-centered interventions. Recruitment challenges and declining retention may signal structural, developmental, and logistical barriers experienced by pediatric survivors and their families. These findings highlight the need for psychosocial oncology interventions to be developmentally informed, flexible in format, and attentive to family systems.

## 6. Staffing Psychosocial Oncology in Cancer Settings

Workforce planning is foundational to the sustainability of psychosocial oncology service delivery. Aligning patient needs with manageable clinical workloads requires accounting not only for direct clinical care but also for administrative and leadership responsibilities, teaching and supervision, research, and committee participation. In the final paper of this Special Issue, Mayer et al. present a psychosocial oncology staffing framework that contributes directly to cancer service planning and system design. Drawing on national consultation with administrators and clinicians that was conducted by the CAPO Clinical Advisory Committee, the paper describes substantial variation across Canadian jurisdictions alongside a lack of clear or consistent rationales for determining psychosocial oncology staffing levels. In response, the authors propose a comprehensive framework to guide the estimation of staffing ratios for psychosocial oncology programming. The implications of this work are important. Without adequate and transparent staffing models, the psychosocial needs of patients with cancer may be systematically under-addressed across cancer care settings, while excessive demands on psychosocial oncology clinicians may increase burnout.

## 7. Looking Forward to the Next Decade of Psychosocial Oncology

The papers in this Special Issue highlight that advancing psychosocial oncology over the coming decade, and beyond, requires more than continued evidence generation. Progress will depend on deliberate system design, sustained investment in the psychosocial oncology workforce, consideration given to novel technologies such as artificial intelligence, and implementation strategies that embed supportive care into routine oncology practice across the cancer continuum. By addressing pragmatic screening, service integration, public understanding of psychosocial oncology, system gaps, and staffing capacity, this Special Issue offers actionable insights for strengthening psychosocial oncology as a core component of high-quality cancer care. At the same time, planning for the future requires a rigorous and comprehensive environmental scan to identify research gaps, emerging priorities, and opportunities for strategic development. The CAPO Research Advisory Committee has identified this as an important next step and plans to address this gap to inform strategic research funding and planning. Together, the contributions of this Special Issue provide a strong foundation for shaping future research priorities and advancing accountable, sustainable integration that is responsive to the evolving needs of patients with cancer.

## Data Availability

Data sharing is not applicable to this article as no new data were created or analyzed.

## Abbreviations

The following abbreviations are used in this manuscript:

CAPO	Canadian Association of Psychosocial Oncology
ESAS	Edmonton Symptom Assessment System
PHQ-9	Patient Health Questionnaire-9
GAD-7	Generalized Anxiety Disorder-7
ACE	Alberta Cancer Exercise
EXCEL	Exercise for Cancer to Enhance Living Well
